# Breastfeeding support provided by lactation consultants in high-income countries for improved breastfeeding rates, self-efficacy, and infant growth: a systematic review and meta-analysis protocol

**DOI:** 10.1186/s13643-023-02239-9

**Published:** 2023-05-02

**Authors:** Curtis J. D’Hollander, Victoria A. McCredie, Elizabeth M. Uleryk, Charles D. G. Keown-Stoneman, Catherine S. Birken, Deborah L. O’Connor, Jonathon L. Maguire

**Affiliations:** 1 Department of Nutritional Sciences, University of Toronto, Toronto, ON Canada; 2 Department of Paediatrics, St. Michael’s Hospital, Toronto, ON Canada; 3 Interdepartmental Division of Critical Care Medicine, University of Toronto, Toronto, ON Canada; 4 Department of Medicine (Respirology), Toronto Western Hospital, University Health Network, Toronto, ON Canada; 5 Institute of Health Policy Management and Evaluation, University of Toronto, Toronto, ON Canada; 6 EM Uleryk Consulting, Mississauga, ON Canada; 7 Applied Health Research Centre, Unity Health Toronto, Toronto, ON Canada; 8 Dalla Lana School of Public Health, University of Toronto, Toronto, ON Canada; 9 Li Ka Shing Knowledge Institute, St. Michael’s Hospital, Toronto, ON Canada; 10 Division of Pediatric Medicine, Hospital for Sick Children, Toronto, ON Canada; 11 Child Health Evaluative Sciences, SickKids Research Institute, Toronto, ON Canada; 12 Department of Pediatrics, Faculty of Medicine, University of Toronto, Toronto, ON Canada; 13 Joannah & Brian Lawson Centre for Child Nutrition, Department of Nutritional Sciences, University of Toronto, Toronto, ON Canada; 14 Translational Medicine Program, Hospital for Sick Children, Toronto, ON Canada

**Keywords:** Lactation consultant, Lactation counselor, Breastfeeding, Self-efficacy, Infant growth, Overweight, Obesity

## Abstract

**Background:**

It is well established that breast milk offers numerous health benefits for mother and child. Mothers are recommended to exclusively breastfeed their child until 6 months of age, with continued breastfeeding up to 1–2 years of age or beyond. Yet, these recommendations are met less than half of the time in high-income countries. Lactation consultants specialize in supporting mothers with breastfeeding and are a promising approach to improving breastfeeding rates. For lactation consultant interventions to be implemented widely as part of public health policy, a better understanding of their effect on breastfeeding rates and important health outcomes is needed.

**Methods:**

The overall aim of this systematic review is to evaluate the effect of lactation consultant interventions provided to women, compared to usual care, on breastfeeding rates (primary outcome), maternal breastfeeding self-efficacy, and infant growth. A search strategy has been developed to identify randomized controlled trials published in any language between 1985 and April 2023 in CENTRAL, MEDLINE, EMBASE, CINAHL, Scopus, and Web of Science. We will also perform a search of the grey literature and reference lists of relevant studies and reviews. Two reviewers will independently extract data on study design, baseline characteristics, details of the interventions employed, and primary and secondary outcomes using a pre-piloted standardized data extraction form. Risk of bias and quality of evidence assessment will be done independently and in duplicate using the Cochrane Risk of Bias tool and GRADE approach, respectively. Where possible, meta-analysis using random-effects models will be performed, otherwise a qualitative summary will be provided. We will adhere to the Preferred Reporting Items for Systematic Reviews and Meta-Analyses (PRISMA) guidelines.

**Discussion:**

This review will fill an important gap in the lactation support literature. The findings will be of importance to policymakers who seek to implement interventions to improve breastfeeding rates.

**Trial registration:**

This review has been registered in the PROSPERO database (ID: CRD42022326597).

**Supplementary Information:**

The online version contains supplementary material available at 10.1186/s13643-023-02239-9.

## Background

Breastmilk is widely accepted as the optimal source of nutrition for infants. The World Health Organization (WHO) and others recommend exclusive breastfeeding until 6 months of age, with continued breastfeeding up to 1–2 years of age or beyond [1–5]. Breastfeeding has been shown to offer numerous health benefits for mother and child. For mothers, it can reduce breast cancer risk, improve birth spacing due to lactational amenorrhea, and may reduce the risk for diabetes [5, 6]. For children, breastfeeding can reduce the risk for obesity, lower infections, and improve neurodevelopment [5–7]. The economic benefit of breastfeeding is substantial. For example, developmental gains due to breastfeeding through 6 months of age were estimated to result in a gain of 0.53% ($231.4 billion US) of high-income countries’ gross national income [8]. Yet, the prevalence of any breastfeeding in high-income countries is only ~ 40% at 6 months, and ~ 20% at 1 year [6]. Scalable interventions to promote breastfeeding are needed [8, 9].

A promising approach to improving breastfeeding rates in high-income countries is lactation consultants (LCs). LCs are experts in supporting breastfeeding and may be certified as an International Board Certified Lactation Consultant (IBCLC), a Certified Lactation Counselor (CLC), or have received other informal training not recognized by a governing agency [10, 11]. A 2017 Cochrane review explored the impact of all forms of breastfeeding support within controlled trials on breastfeeding rates. The results showed the benefit of offering breastfeeding support to women but did not distinguish the effect of LCs from other health professionals [12]. A 2016 systematic review explored the impact of breastfeeding support provided by LCs or lactation counselors within randomized controlled trials (RCTs) on breastfeeding rates. It was found that LCs improved breastfeeding rates, but the review did not account for the certification of the LCs and did not perform any subgroup analyses to elucidate the key components of effective interventions [13]. A 2019 systematic review examined the impact of support provided by IBCLCs within pre-post, randomized, and non-randomized trials on breastfeeding rates. The results showed IBCLCs to have beneficial effects on breastfeeding; however, this review was limited in scope to interventions provided in-person by IBCLCs during the postpartum period and did not consider outcomes beyond breastfeeding prevalence [14]. Furthermore, newer studies have emerged, allowing for a better understanding of maternal and infant outcomes. For instance, Linares et al. [15] conducted a RCT which compared an intervention consisting of a home visit provided by a LC to usual care on breastfeeding outcomes. Cauble et al. [16] conducted a randomized controlled pilot trial which compared an intervention consisting of group based phone counselling provided by a LC to usual care on breastfeeding outcomes. The present systematic review will address the aforementioned gaps and include newer studies to inform effective breastfeeding support interventions for public health policy.

### Objectives

This systematic review aims to understand the effect of interventions provided by LCs to improve breastfeeding rates. The primary objective of this study is to determine whether women who receive support from a LC, compared to women who receive usual care, have a lower number who stop exclusive breastfeeding before 6 months. This will include women who may decide to breastfeed or are currently breastfeeding. Secondary objectives are to determine among women, whether receiving support from a LC, compared to usual care, has a beneficial effect on (1) the number of women who stop any breastfeeding before 6 months, (2) the number of women who stop exclusive breastfeeding before 4–6 weeks, (3) the number of women who stop any breastfeeding before 4–6 weeks, (4) exclusive breastfeeding duration, (5) any breastfeeding duration, (6) maternal breastfeeding self-efficacy, (7) infant overweight/obesity, and (8) infant growth.

We will also aim to identify the characteristics and participants of interventions that were most effective for the primary objective with meta-regression models. This includes income of participants, parity, maternal overweight/obesity, timing of intervention, in-person or virtual consults, breastfeeding supplies provided, certification of LC, intensity of intervention, gestational age, and individual or group support.

## Methods

### Design

This protocol follows the Preferred Reporting Items for Systematic Review and Meta-Analysis Protocols 2015 (PRISMA-P) (see Additional file 1) [17]. Additionally, the guidelines set out in the Cochrane Handbook for Systematic Reviews of Interventions will be followed [18]. This protocol was registered in the PROSPERO database (https://www.crd.york.ac.uk/prospero/). Should an amendment to the protocol be required, we will document the date of the change, details of the amendment, and rationale within PROSPERO. We will also note the amendment(s) within the final manuscript of results.

### Eligibility criteria

We will apply the following eligibility criteria to identify studies for inclusion in this review.

### Study design

We will include RCTs, also incorporating cluster RCTs, performed in high-income countries as classified by the World Bank [19].

### Population of interest

Women (including preconception and pregnant women) who may decide to breastfeed or are currently breastfeeding their infant. Infants must be born late preterm (≥ 34 week’s gestation) or term (≥ 37 week’s gestation).

### Intervention

All interventions delivered directly by LCs with the intent to improve breastfeeding which is beyond usual care. As LCs can have various certifications and training backgrounds, LCs to provide the intervention and be included in the review are detailed in Table 1. We will consider interventions which are provided at any time (i.e., preconception, prenatally, perinatally, postnatally); provided solely by LCs or as part of a multi-component intervention; in-person or remote; individual or group support; provided as counselling, education, or training; delivered to the mother or others (e.g., healthcare team, father, caregivers); provided once or repeatedly; offered proactively or reactively. Interventions which include any combination of these characteristics will be included.

**Table 1:** Definition of lactation consultants to provide the intervention

Type	Definition
International Board Certified Lactation Consultant (IBCLC)	IBCLC’s are certified with the International Board of Lactation Consultant Examiners and have undergone health sciences education, lactation specific education, relevant clinical experience, and passed the IBCLC examination [10]
Certified Lactation Counselor (CLC)	CLC’s are required to complete lactation training, demonstrate competency in supporting women with breastfeeding, and passed the CLC examination [11]
Lactation Consultant or Lactation Counselor	If the study mentions the intervention was provided by a “Lactation Consultant” or “Lactation Counselor”, whether or not they specify additional training or certification

### Comparators

The comparator is usual care as defined within each study. It is expected that usual breastfeeding care will consist of community resources which may be proactively sought by participants (e.g., community groups, public health, private practice), and standard in-hospital support. If the comparator is offering an intervention beyond usual care, then the study will be excluded.

### Outcomes

Our primary outcome will be the number of women who stop exclusive breastfeeding before 6 months. Secondary outcomes of interest include.

- 1. Number of women who stop any breastfeeding before 6 months.
- 2. Number of women who stop exclusive breastfeeding before 4–6 weeks.
- 3. Number of women who stop any breastfeeding before 4–6 weeks.
- 4. Exclusive breastfeeding duration.
- 5. Any breastfeeding duration.
- 6. Maternal breastfeeding self-efficacy.
- 7. Infant overweight/obesity.
- 8. Infant growth.

The breastfeeding outcomes were selected to align with the primary outcomes used in recent breastfeeding support Cochrane reviews and similar meta-analyses, and to allow for easy comparison [12, 20, 21]. Furthermore, the primary outcome was selected to align with the current WHO exclusive breastfeeding recommendation. The WHO defines exclusive breastfeeding as consuming nothing but breastmilk (including expressed breastmilk), with the exception of vitamins, minerals, and medicines [22]. We anticipate some studies using a different definition of exclusive breastfeeding. We will follow the definition used in each study and document the definition used. Any breastfeeding will be considered consuming any breastmilk, including exclusive breastfeeding and breastfeeding while consuming other liquids and solid foods. For the breastfeeding outcomes, the time at the last study assessment will be used up to 6 months or 4–6 weeks, as well as the longest duration of exclusive or any breastfeeding measured on a continuous scale in months. Breastmilk fed at the breast or bottle fed will be included.

Maternal breastfeeding self-efficacy is a modifiable factor which could help explain any observed changes in breastfeeding rates. Only studies which used a validated breastfeeding self-efficacy tool will be considered. For example, the validated 33 item Breastfeeding Self-efficacy Scale (BSES) and the 14 item BSES-Short Form (BSES-SF) are measured on a continuous scale and commonly used [23, 24]. Should breastfeeding self-efficacy measurements be taken at multiple time points, we will use the measurement taken closest to 1 month postpartum, but not before 2 weeks or after 6 weeks. Previous systematic reviews have shown 1 month postpartum to be when studies typically report breastfeeding self-efficacy and is within the age range of when most tools are validated [25, 26].

Infant overweight and obesity will be assessed as one category, regardless of the measurement used within the studies, as defined by the WHO, Centers for Disease Control and Prevention (CDC), International Obesity Task Force (IOTF), or study authors [27–29]. For example, based on the WHO growth standards, overweight is defined as a body mass index *z*-score > 2 and obesity is defined as a body mass index *z*-score > 3 (for children ≤ 5 years) [30]. Infant growth will include all continuous growth measures. This includes body mass index, weight for age, body fat mass, lean body mass, weight, height, waist circumference, waist-to-hip ratio, body fat percentage, skinfold thickness, and *z*-scores (based on any reference population). The measure at the last study assessment will be used.

### Information sources and search strategy

Databases to be searched include the Cochrane Central Register of Controlled Trials (CENTRAL), MEDLINE (Ovid), EMBASE (Ovid), Cumulative Index to Nursing and Allied Health Literature (CINAHL) (EBSCO), Scopus, and Web of Science. Scopus and Web of Science will be searched only for conference papers and abstracts. We will also perform a search of the grey literature to identify unpublished and other studies. This includes searching regulatory agency sources, OpenAIRE, and hand searching reference lists of included studies and relevant reviews. The search strategy was developed in conjunction with a health information specialist (EU). The strategy includes derivations of the following: Breastfeed* OR Lactation AND Consult* OR Counsel* AND Educat* OR Support*. The Cochrane sensitive RCT filter was used and a limit was added for publication year [18, 31]. The search will be limited to sources from 1985 (when IBCLCs were founded) until April 2023. The search strategy for each of the databases can be found in Additional file 2. There will be no restriction on study language, and translation will be performed in Google Translate, as required. The search will be re-run in each database prior to publication to identify and include new studies.

### Study selection

All study titles and abstracts retrieved through the search will be uploaded and managed within the Covidence software (Veritas Health Innovation, Melbourne, Australia). First, duplicates will be removed and titles and abstracts will be screened against the eligibility criteria independently by two reviewers. The application of the eligibility criteria will be pilot tested at the start of title and abstract screening among reviewers with six studies to ensure the eligibility criteria is applied consistently and clarification is provided, as needed. Studies which may meet eligibility criteria, as judged by either reviewer, will undergo full text review. Full-text review will also be done independently by two reviewers. Disagreement between reviewers will be resolved through discussion and reasons for excluding studies at the full text stage will be documented and included in the final published manuscript. Should clarification be needed, we will email corresponding authors for further information. If there is no response, we will send one follow-up email. This will result in a list of studies for data extraction.

### Data extraction

Data extraction will be done independently by two reviewers using a pre-piloted data extraction form to ensure consistency across reviewers. The extraction form has been piloted on three studies known to meet eligibility criteria (see Additional file 3). Data extracted will include study information, study characteristics, and description of participants. Details of the intervention, comparators, and the outcomes of interest including adherence will also be extracted. Disagreement on data extraction between reviewers will be resolved with discussion. Study authors will be emailed a maximum of two times (i.e., initial and follow-up) for clarification or further information, if needed. Multiple reports of the same study will be identified through study characteristics (e.g., trial registration number, author names, sponsorship, location/setting, intervention, participants, date/duration of study, length of follow-up, subgroups). If uncertainty remains, author(s) will be contacted. Infants of mothers included in the studies will be assumed to be born full term if not explicitly mentioned.

### Assessment of methodological quality and risk of bias

Eligible RCTs will be assessed using version two of the Cochrane risk of bias tool for randomized trials which includes additional guidance for cluster RCTs [18, 32]. The five bias domains within the tool which will be assessed include bias from randomization process, deviation from intended interventions, missing outcome data, measurement of outcome, and selection of results reported. This will be assessed for study outcomes reported in the summary of findings table. Risk of bias will be judged to be “low”, “some concerns”, or “high” with an explanation for each domain. Risk of bias assessment will be done independently by two reviewers. Reviewers will not be blinded to studies and disagreements will be resolved by discussion. Study authors will be contacted if additional information is required for the assessment. No studies will be excluded based on their risk of bias assessment. Risk of bias assessment will be displayed for each study outcome and cumulatively across the domains. Sensitivity analysis will be performed for studies deemed low risk of bias. This will help in determining whether the results are robust, identifying possible bias and strengthening conclusions. Meta-biases for reporting bias and publication bias will be assessed. Protocols of RCTs will be checked to determine if outcome reporting bias was present. When a protocol is unavailable, a comparison will be made between the reporting in the methods and results section. Funnel plots will be constructed and visually inspected to detect publication bias if ≥ 10 studies are included in the meta-analysis.

### Data synthesis and analysis

Data extracted will be reported in descriptive tables to illustrate important study characteristics. Continuous data will be reported as means with standard deviations and categorical data as counts and percentages. If studies report the median and range only, the method developed by McGrath et al. will be used to estimate the mean and SD [33]. Pooled estimates of effect sizes will be presented as risk ratios with 95% confidence intervals (CIs) for dichotomous variables. Continuous variables will be presented as a mean difference (MD) with 95% CIs. When different measurement scales are used, a standardized MD with 95% CIs will be presented. If there are at least two studies for an outcome, meta-analyses will be conducted using random-effects models and inverse variance weighting with the Paule-Mandel procedure because heterogeneity is expected in terms of interventions and populations studied [34, 35]. Estimation of a confidence interval for the between-study variation will be done using the Q-Profile method [34, 36]. Heterogeneity will be tested using the $\chi$ ^2^ test and I^2^ test. For $\chi$ ^2^, *P* < 0.10 will be considered significant heterogeneity. For *I*^2^, heterogeneity will be classified as negligible (< 40%), moderate (30–60%), substantial (50–90%), or considerable (75–100%) [18]. The variation across studies will be described with $\tau$ ^2^. 95% prediction intervals will be constructed around the overall effects which accounts for between study heterogeneity.

Results from intention to treat analyses will be used, when possible. If data are missing, we will contact study author(s) and document the level of incomplete results. Forest plots will be used to display results. If a study has > 2 groups (i.e., multiple interventions), the intervention groups will be combined to create a single pairwise comparison with the control group, if possible. Otherwise, only the intervention group which most closely aligns with the eligibility criteria will be used. For cluster RCTs which have not been analyzed appropriately (e.g., unit-of-analysis error), they will be adjusted for using the formula described in the Cochrane handbook to reduce the size of each trial to its effective sample size [18]. The effective sample size is the original sample size divided by the design effect. The design effect is calculated with the formula: 1 + (average cluster size − 1) $\times$ intracluster correlation coefficient (ICC). If an estimate of the ICC is unavailable, it will be estimated from a similar study.

If there are insufficient studies, meta-analysis will not be performed, and a narrative summary will be provided with studies described in text and tables. Statistical analysis will be performed using R software version 4.1.2 or greater [37].

### Meta-regression and sensitivity analyses

We have identified several clinically meaningful meta-regression and sensitivity analyses a priori which will be performed if sufficient data is available, restricted to the primary outcome. These will allow for a better understanding of sources of heterogeneity and characteristics of effective interventions. Sensitivity analysis will be performed for studies with low risk of bias. This will be achieved by re-running the meta-analysis among only studies judged to be low risk of bias from the Cochrane risk of bias tool and comparing the results to the meta-analysis with all the studies. Meta-regression will be performed if there are at least 10 studies. Meta-regression analyses will be done for the following characteristics:

- 1. Income of participants (low vs. mixed income as defined within each study. Studies with > 75% of participants with low-income will be categorized as low-income).
- 2. Parity (primiparous vs. primiparous and multiparous).
- 3. Maternal overweight/obesity (healthy weight vs. overweight or obese as defined by study authors based upon pre-pregnancy body mass index (BMI)).
- 4. Timing of intervention (prenatal vs. postnatal vs. both).
- 5. Provision of intervention (in-person vs. virtual vs. both).
- 6. Breastfeeding supplies provided during intervention (breast pump provided vs. not provided).
- 7. Certification of LC (IBCLC or CLC vs. other).
- 9. Intensity of intervention (number of contacts, continuous).
- 9. Intervention delivery (individual vs. group vs. both).
- 10. Gestational age (term vs. preterm and term).
- 11. Publication year (continuous).

### Assessing the quality of evidence

The quality of evidence will be assessed independently by two reviewers for stopping exclusive breastfeeding before 6 months and 4–6 weeks, stopping any breastfeeding before 6 months and 4–6 weeks, and exclusive and any breastfeeding duration. This will be done using the Grading of Recommendations Assessment, Development, and Evaluation (GRADE) approach [38]. Items to be considered include 5 domains: risk of bias, inconsistency, indirectness, imprecision, and publication bias. This will yield an assessment of the certainty of evidence as high, moderate, low, or very low and will be presented within a summary of findings table.

## Discussion

This systematic review will assess the effect of LC interventions on breastfeeding rates, breastfeeding self-efficacy, and infant growth. Lactation support interventions have been shown to be effective, but the effectiveness and characteristics of interventions provided by LCs are not well defined. Specifically, we will illuminate the characteristics of LCs which are most effective, include pre and postnatal interventions, and maternal and infant health outcomes.

With the benefits of breastfeeding being numerous, and accessible to nearly all women, interventions are needed to improve breastfeeding rates. The findings of this systematic review will describe the effectiveness of LC interventions and elucidate their key characteristics. This will be of importance to policy makers designing interventions to improve breastfeeding rates.

## Supplementary Information

**Additional file 1.** PRISMA-P checklist.

**Additional file 2.** Search strategy.

**Additional file 3.** Data extraction form.

## Data Availability

Not applicable.

## Abbreviations

BMI: Body mass index
CDC: Centers for Disease Control and Prevention
CI: Confidence interval
CLC: Certified Lactation Counselor
IBCLC: International Board Certified Lactation Consultant
ICC: Intracluster correlation coefficient
LC: Lactation Consultant
MD: Mean difference
RCT: Randomized controlled trial
WHO: World Health Organization
