# Supplementary material for: Breastfeeding support provided by lactation consultants in high-income countries for improved breastfeeding rates, self-efficacy, and infant growth: a systematic review and meta-analysis protocol
Source: Syst Rev. 2023 May 2;12:75. doi: 10.1186/s13643-023-02239-9 (PMC10152596; doi:10.1186/s13643-023-02239-9)
Supplement: Supplementary file 2 — Additional file 2. Search strategy. [file 13643_2023_2239_MOESM2_ESM.docx]

**Additional File 2: Search strategy**

1. MEDLINE (Ovid)

| **#** | **Searches** |
| --- | --- |
| 1 | (lactation adj2 (consult* or counsel*)).ti,ab,kf. |
| 2 | lactation/ or milk ejection/ |
| 3 | breast feeding/ or breast milk expression/ |
| 4 | MILK, HUMAN/ |
| 5 | (breastfeed* or breastfed* or (breast adj2 feed*) or lactat* or breast-feed* or "breast-fed" or (breast adj2 fed)).ti,ab,kf,jn. |
| 6 | or/2-5 |
| 7 | Consultants/ |
| 8 | Counseling/ or Directive Counseling/ or Distance Counseling/ |
| 9 | (consultant* or trainer* or teach* or instruct* or educat* or counsel* or support* or special*).ti,ab,kf. |
| 10 | or/7-9 |
| 11 | 1 or (6 and 10) |
| 12 | education/ or curriculum/ or competency-based education/ or interdisciplinary studies/ or problem-based learning/ or education, professional/ or health education/ or consumer health information/ or health fairs/ or health promotion/ or patient education as topic/ or education, distance/ or prenatal education/ |
| 13 | "attitude of health personnel"/ or health knowledge, attitudes, practice/ |
| 14 | Telemedicine/ |
| 15 | Community health services/ or Maternal health services/ or maternal-child health services/ or perinatal care/ or preconception care/ or prenatal care/ or postnatal care/ |
| 16 | social support/ |
| 17 | "Standard of Care"/ |
| 18 | (board adj2 certif*).ti,ab,kf. |
| 19 | or/12-18 |
| 20 | 11 and 19 |
| 21 | randomized controlled trial.pt. |
| 22 | controlled clinical trial.pt. |
| 23 | randomized.ab. |
| 24 | placebo.ab. |
| 25 | drug therapy.fs. |
| 26 | randomly.ab. |
| 27 | trial.ab. |
| 28 | groups.ab. |
| 29 | or/21-28 |
| 30 | exp animals/ not humans.sh. |
| 31 | 29 not 30 |
| 32 | Comparative Effectiveness Research/ |
| 33 | intention to treat analysis/ or pragmatic clinical trials as topic/ |
| 34 | Multicenter Studies as Topic/ |
| 35 | (comparative study or multicenter study or pragmatic clinical trial).pt. |
| 36 | 31 or 32 or 33 or 34 or 35 |
| 37 | 20 and 36 |
| 38 | limit 37 to yr="1985-Current" |

Line 31 is the Cochrane Handbook Highly Sensitive Search Strategy for identifying randomized trials in MEDLINE (Box 6.4.c 2008 version)

1. EMBASE (Ovid)

Embase Classic+Embase

| **#** | **Searches** |
| --- | --- |
| 1 | lactation consultant/ |
| 2 | (lactation adj2 consultant*).ti,ab,kf,jn. |
| 3 | lactation/ or exp lactation disorder/ or exp lactation inhibition/ |
| 4 | breast feeding/ or breast milk expression/ or breast feeding education/ |
| 5 | or/3-4 |
| 6 | consultation/ |
| 7 | (consultant* or consulting or consulted or train* or educat* or instruct*).ti,ab,kf. |
| 8 | or/6-7 |
| 9 | 1 or 2 or (5 and 8) |
| 10 | education/ or curriculum development/ or education program/ or educational model/ or problem based learning/ or teaching/ |
| 11 | health education/ or breast feeding education/ or childbirth education/ or parenting education/ or patient education/ |
| 12 | (board adj2 certif*).ti,ab,kf. |
| 13 | certification/ |
| 14 | or/10-13 |
| 15 | 9 and 14 |
| 16 | randomized controlled trial/ |
| 17 | Controlled clinical study/ |
| 18 | random*.ti,ab. |
| 19 | Randomization/ |
| 20 | Intermethod comparison/ |
| 21 | placebo*.ti,ab. |
| 22 | (compare or compared or comparison).ti. |
| 23 | ((evaluated or evaluate or evaluating or assessed or assess) and (compare or compared or comparing or comparison)).ab. |
| 24 | (open adj label).ti,ab. |
| 25 | ((double or single or doubly or singly) adj (blind or blinded or blindly)).ti,ab. |
| 26 | double-blind procedure/ |
| 27 | Parallel group$1.ti,ab. |
| 28 | (Crossover or cross over).ti,ab. |
| 29 | ((assign* or match or matched or allocation) adj5 (alternate or group$1 or intervention$1 or patient$1 or subject$1 or participant$1)).ti,ab. |
| 30 | (assigned or allocated).ti,ab. |
| 31 | (controlled adj7 (study or design or trial)).ti,ab. |
| 32 | (volunteer or volunteers).ti,ab. |
| 33 | Human experiment/ |
| 34 | Trial.ti. |
| 35 | or/16-34 |
| 36 | (Random$ adj sample$ adj7 ("cross section$" or questionnaire$1 or survey$1 or database$1)).ti,ab. not (comparative study/ or controlled study/ or randomi$ed controlled.ti,ab. or randomly assigned.ti,ab.) |
| 37 | Cross-sectional study/ not (randomized controlled study/ or controlled clinical study/ or controlled study/ or randomi?ed controlled.ti,ab. or control groups$1.ti,ab.) |
| 38 | (((case adj control$) and random$) not randomi?ed controlled).ti,ab. |
| 39 | (Systematic review not (trial or study)).ti. |
| 40 | (nonrandom$ not random$).ti,ab. |
| 41 | "random field$".ti,ab. |
| 42 | (random cluster adj3 sampl$).ti,ab. |
| 43 | (review.ab. and review.pt.) not trial.ti. |
| 44 | ("we searched".ab. and review.ti.) or review.pt. |
| 45 | "update review".ab. |
| 46 | (databases adj4 searched).ab. |
| 47 | (rat or rats or mouse or mice or swine or porcine or murine or sheep or lambs or pigs or piglets or rabbit or rabbits or cat or cats or dog or dogs or cattle or bovine or monkey or monkeys or trout or marmoset$1).ti. and animal experiment/ |
| 48 | Animal experiment/ not (human experiment/ or human/) |
| 49 | or/36-48 |
| 50 | 35 not 49 |
| 51 | 15 and 50 |
| 52 | limit 51 to yr="1985 -Current" |

Line 50 is Cochrane Box 3.e EMBASE sensitive Therapy Treatment Effectiveness Filter terms 2018 revision (Glanville et al 2019b)

1. CINAHL Plus with Full Text (Ebsco)

| **Search ID #** | **Search Terms** |
| --- | --- |
| S46 | S21 AND S44  [Limiters - Published Date: 19850101-20230430] |
| S45 | S21 AND S44 |
| S44 | S43 NOT S42 |
| S43 | S22 OR S23 OR S24 OR S25 OR S26 OR S27 OR S28 OR S29 OR S30 OR S31 OR S32 OR S33 OR S34 OR S35 OR S36 |
| S42 | S40 NOT S41 |
| S41 | MH (human) |
| S40 | S37 OR S38 OR S39 |
| S39 | TI (animal model*) |
| S38 | MH (animal studies) |
| S37 | MH animals+ |
| S36 | AB (cluster W3 RCT) |
| S35 | MH (crossover design) OR MH (comparative studies) |
| S34 | AB (control W5 group) |
| S33 | PT (randomized controlled trial) |
| S32 | MH (placebos) |
| S31 | MH (sample size) AND AB (assigned OR allocated OR control) |
| S30 | TI (trial) |
| S29 | AB (random*) |
| S28 | TI (randomised OR randomized) |
| S27 | MH cluster sample |
| S26 | MH pretest-posttest design |
| S25 | MH random assignment |
| S24 | MH single-blind studies |
| S23 | MH double-blind studies |
| S22 | MH randomized controlled trials |
| S21 | S12 and S20 |
| S20 | S13 OR S14 OR S15 OR S16 OR S17 OR S18 OR S19 |
| S19 | (board N2 (certif*)) |
| S18 | (MH "Allied Health Personnel") |
| S17 | (MH "Maternal-Child Care+") |
| S16 | (MH "Patient Education") OR (MH "Health Education") OR (MH "Nutrition Education") |
| S15 | (MH "Health Promotion") OR (MH "Breast Feeding Promotion") |
| S14 | (MH "Telemedicine") OR (MH "Remote Consultation") OR (MH "Telenutrition") |
| S13 | (MH "Education+") |
| S12 | S6 OR (S5 AND S11) |
| S11 | S7 OR S8 OR S9 OR S10 |
| S10 | (consultant* OR trainer* OR teach* OR instruct* OR educat* OR counsel* OR support* OR special*) |
| S9 | (MH "Counseling+") |
| S8 | (MH "Counselors") OR (MH "Consultants") |
| S7 | (MH "Lactation Consultants") |
| S6 | (lactation N2 (consult* OR counsel*)) |
| S5 | S1 OR S2 OR S3 OR S4 |
| S4 | (MH "Infant Nutrition+") OR (MH "Breast Feeding") OR (MH "Weaning") OR (MH "Milk, Human+") |
| S3 | (MH "Lactation") |
| S2 | (breast N2 (feed*)) OR (breast N2 (fed)) |
| S1 | (breastfeed* OR breastfed* OR lactat* OR "breast-feed" OR "breast-fed") |

1. Cochrane Central Register of Controlled Trials (CENTRAL)

| **ID** | **Search** |
| --- | --- |
| #1 | lactation NEAR/2 (consult* or counsel*):ti,ab,kw |
| #2 | [mh ^"Lactation"] OR [mh ^"Milk Ejection"] |
| #3 | [mh ^"Breastfeeding"] OR [mh ^"Breast Milk Expression"] |
| #4 | [mh ^"Human Milk"] |
| #5 | (breastfeed* or breastfed* or (breast NEAR/2 feed*) or lactat* or breast-feed* or "breast-fed" or (breast NEAR/2 fed)):ti,ab,kw |
| #6 | {OR #2-#5} |
| #7 | [mh ^"Consultants"] |
| #8 | [mh ^"Counseling"] OR [mh ^"Directive Counseling"] OR [mh ^"Distance Counseling"] |
| #9 | (consultant* OR trainer* OR teach* OR instruct* OR educat* OR counsel* OR support* OR special*):ti,ab,kw |
| #10 | {OR #7-#9} |
| #11 | #1 OR (#6 AND #10) |
| #12 | [mh ^"education"] OR [mh ^"curriculum"] OR [mh ^"competency-based education"] OR [mh ^"interdisciplinary studies"] OR [mh ^"problem-based learning"] OR [mh ^"education, professional"] OR [mh ^"health education"] OR [mh ^"consumer health information"] OR [mh ^"health fairs"] OR [mh ^"health promotion"] OR [mh ^"patient education as topic"] OR [mh ^"education, distance"] OR [mh ^"prenatal education"] |
| #13 | [mh ^"attitude of health personnel"] OR [mh ^"health knowledge, attitudes, practice"] |
| #14 | [mh ^"Telemedicine"] |
| #15 | [mh ^"Community health services"] OR [mh ^"Maternal health services"] OR [mh ^"maternal-child health services"] OR [mh ^"perinatal care"] OR [mh ^"preconception care"] OR [mh ^"prenatal care"] OR [mh ^"postnatal care"] |
| #16 | [mh ^"social support"] |
| #17 | [mh ^"Standard of Care"] |
| #18 | (board NEAR/2 certif*):ti,ab,kw |
| #19 | {OR #12-#18} |
| #20 | #11 AND #19  with Publication Year from 1985 to 2023, with Cochrane Library publication date between Jan 1985 and Apr 2023, in Trials |
| #21 | #11 AND #19  with Publication Year from 1985 to 2023, with Cochrane Library publication date between Jan 1985 and Apr 2023, in Trials |

1. Scopus

| **Search** |
| --- |
| ( ( ( TITLE-ABS-KEY ( lactation  W/2  ( consult*  OR  counsel* ) ) )  OR  ( ( TITLE-ABS-KEY ( breastfeed*  OR  breastfed*  OR  ( breast  W/2  feed* )  OR  lactat*  OR  breast-feed*  OR  "breast-fed"  OR  ( breast  W/2  fed ) ) )  AND  ( TITLE-ABS-KEY ( consultant*  OR  trainer*  OR  teach*  OR  instruct*  OR  educat*  OR  counsel*  OR  support*  OR  special* ) ) ) )  AND  ( TITLE-ABS-KEY ( educat*  OR  learn*  OR  program*  OR  activit*  OR  workshop*  OR  support*  OR  curriculum*  OR  course*  OR  stud*  OR  campaign*  OR  promot*  OR  telemedicine*  OR  telehealth  OR  ehealth  OR  train*  OR  service*  OR  care  OR  ( standard  W/2  care )  OR  ( board  W/2  certif* ) ) ) )  AND  ( TITLE-ABS-KEY ( {Clinical-trial}  OR  {controlled-trial}  OR  randomi*  OR  randomly  OR  ( random  W/4  ( allocat*  OR  distribut*  OR  assign* ) )  OR  {placebo}  OR  {trial}  OR  {groups}  OR  {subgroups} )  OR  TITLE ( rct ) )  AND  PUBYEAR  >  1984  AND  ( LIMIT-TO ( DOCTYPE ,  "cp" ) ) |

1. Web of Science (Clarivate)

| **ID** | **Search** |
| --- | --- |
| 12 | **#11 AND #10** |
| 11 | **DT=(Meeting Abstract OR Proceedings Paper)** |
| 10 | **#9 AND #8** |
| 9 | **PY=(1985-2023)** |
| 8 | #7 AND #6 |
| 7 | TS=(randomised OR randomized OR randomisation OR randomisation OR placebo* OR (random* AND (allocat* OR assign*) ) OR (blind* AND (single OR double OR treble OR triple) ) ) NOT TS=(animal or animals or pisces or fish or fishes or catfish or catfishes or sheatfish or silurus or arius or heteropneustes or clarias or gariepinus or fathead minnow or fathead minnows or pimephales or promelas or cichlidae or trout or trouts or char or chars or salvelinus or salmo or oncorhynchus or guppy or guppies or millionfish or poecilia or goldfish or goldfishes or carassius or auratus or mullet or mullets or mugil or curema or shark or sharks or cod or cods or gadus or morhua or carp or carps or cyprinus or carpio or killifish or eel or eels or anguilla or zander or sander or lucioperca or stizostedion or turbot or turbots or psetta or flatfish or flatfishes or plaice or pleuronectes or platessa or tilapia or tilapias or oreochromis or sarotherodon or common sole or dover sole or solea or zebrafish or zebrafishes or danio or rerio or seabass or dicentrarchus or labrax or morone or lamprey or lampreys or petromyzon or pumpkinseed or pumpkinseeds or lepomis or gibbosus or herring or clupea or harengus or amphibia or amphibian or amphibians or anura or salientia or frog or frogs or rana or toad or toads or bufo or xenopus or laevis or bombina or epidalea or calamita or salamander or salamanders or newt or newts or triturus or reptilia or reptile or reptiles or bearded dragon or pogona or vitticeps or iguana or iguanas or lizard or lizards or anguis fragilis or turtle or turtles or snakes or snake or aves or bird or birds or quail or quails or coturnix or bobwhite or colinus or virginianus or poultry or poultries or fowl or fowls or chicken or chickens or gallus or zebra finch or taeniopygia or guttata or canary or canaries or serinus or canaria or parakeet or parakeets or grasskeet or parrot or parrots or psittacine or psittacines or shelduck or tadorna or goose or geese or branta or leucopsis or woodlark or lullula or flycatcher or ficedula or hypoleuca or dove or doves or geopelia or cuneata or duck or ducks or greylag or graylag or anser or harrier or circus pygargus or red knot or great knot or calidris or canutus or godwit or limosa or lapponica or meleagris or gallopavo or jackdaw or corvus or monedula or ruff or philomachus or pugnax or lapwing or peewit or plover or vanellus or swan or cygnus or columbianus or bewickii or gull or chroicocephalus or ridibundus or albifrons or great tit or parus or aythya or fuligula or streptopelia or risoria or spoonbill or platalea or leucorodia or blackbird or turdus or merula or blue tit or cyanistes or pigeon or pigeons or columba or pintail or anas or starling or sturnus or owl or athene noctua or pochard or ferina or cockatiel or nymphicus or hollandicus or skylark or alauda or tern or sterna or teal or crecca or oystercatcher or haematopus or ostralegus or shrew or shrews or sorex or araneus or crocidura or russula or european mole or talpa or chiroptera or bat or bats or eptesicus or serotinus or myotis or dasycneme or daubentonii or pipistrelle or pipistrellus or cat or cats or felis or catus or feline or dog or dogs or canis or canine or canines or otter or otters or lutra or badger or badgers or meles or fitchew or fitch or foumart or foulmart or ferrets or ferret or polecat or polecats or mustela or putorius or weasel or weasels or fox or foxes or vulpes or common seal or phoca or vitulina or grey seal or halichoerus or horse or horses or equus or equine or equidae or donkey or donkeys or mule or mules or pig or pigs or swine or swines or hog or hogs or boar or boars or porcine or piglet or piglets or sus or scrofa or llama or llamas or lama or glama or deer or deers or cervus or elaphus or cow or cows or bos taurus or bos indicus or bovine or bull or bulls or cattle or bison or bisons or sheep or sheeps or ovis aries or ovine or lamb or lambs or mouflon or mouflons or goat or goats or capra or caprine or chamois or rupicapra or leporidae or lagomorpha or lagomorph or rabbit or rabbits or oryctolagus or cuniculus or laprine or hares or lepus or rodentia or rodent or rodents or murinae or mouse or mice or mus or musculus or murine or woodmouse or apodemus or rat or rats or rattus or norvegicus or guinea pig or guinea pigs or cavia or porcellus or hamster or hamsters or mesocricetus or cricetulus or cricetus or gerbil or gerbils or jird or jirds or meriones or unguiculatus or jerboa or jerboas or jaculus or chinchilla or chinchillas or beaver or beavers or castor fiber or castor canadensis or sciuridae or squirrel or squirrels or sciurus or chipmunk or chipmunks or marmot or marmots or marmota or suslik or susliks or spermophilus or cynomys or cottonrat or cottonrats or sigmodon or vole or voles or microtus or myodes or glareolus or primate or primates or prosimian or prosimians or lemur or lemurs or lemuridae or loris or bush baby or bush babies or bushbaby or bushbabies or galago or galagos or anthropoidea or anthropoids or simian or simians or monkey or monkeys or marmoset or marmosets or callithrix or cebuella or tamarin or tamarins or saguinus or leontopithecus or squirrel monkey or squirrel monkeys or saimiri or night monkey or night monkeys or owl monkey or owl monkeys or douroucoulis or aotus or spider monkey or spider monkeys or ateles or baboon or baboons or papio or rhesus monkey or macaque or macaca or mulatta or cynomolgus or fascicularis or green monkey or green monkeys or chlorocebus or vervet or vervets or pygerythrus or hominoidea or ape or apes or hylobatidae or gibbon or gibbons or siamang or siamangs or nomascus or symphalangus or hominidae or orangutan or orangutans or pongo or chimpanzee or chimpanzees or pan troglodytes or bonobo or bonobos or pan paniscus or gorilla or gorillas or troglodytes) |
| 6 | **#5 AND #4** |
| 5 | **TS=(Educat* OR learn* OR program* OR workshop* OR support* OR curriculum* OR course* OR campaign* OR promot* OR telemedicine* OR telehealth OR ehealth OR train* OR service* OR (standard NEAR/2 care) OR (board NEAR/2 certif*))** |
| 4 | **#1 OR (#2 AND #3)** |
| 3 | **TS=(consultant* OR trainer* OR teach* OR instruct* OR educat* OR counsel* OR support* OR special*)** |
| 2 | TS=(breastfeed* OR breastfed* OR ( breast NEAR/2 feed* ) OR lactat* OR breast-feed* OR "breast-fed" OR ( breast NEAR/2 fed ) ) |
| 1 | **TS=(lactation NEAR/2 (consult* OR counsel*))** |

1. OpenAIRE EXPLORE

| **Search** |
| --- |
| Search for derivations of: Breastfeed* OR Lactation AND Consult* OR Counsel* AND Educat* OR Support* in “Research products” limited to year range “1985-2023” |
