# Supplementary material for: Breastfeeding support provided by lactation consultants in high-income countries for improved breastfeeding rates, self-efficacy, and infant growth: a systematic review and meta-analysis protocol
Source: Syst Rev. 2023 May 2;12:75. doi: 10.1186/s13643-023-02239-9 (PMC10152596; doi:10.1186/s13643-023-02239-9)
Supplement: Supplementary file 3 — Additional file 3. Data extraction form. [file 13643_2023_2239_MOESM3_ESM.docx]

**Additional File 3: Data extraction form**

Version 1.0 March 27, 2023 – adapted for use in Covidence

| **Information about data extraction from reports** | |
| --- | --- |
| Data extractor |  |
| Date of data extraction |  |
| Report title |  |
| Year of publication |  |
| First author (last name) |  |
| Author’s contact details (email) |  |
| Author(s) contacted (if yes, specify the reason): | |
| Notes: | |

| **Eligibility criteria**  RCTs (not quasi) of interventions provided by lactation consultants (LCs) to improve breastfeeding, compared to usual care. Participants include women (with child born ≥34 week’s gestation, if applicable) and high-income countries only. |
| --- |
| Confirm eligibility criteria met.  INCLUDE  EXCLUDE **** |
| If exclude, provide reason:   Not a RCT  No LC intervention  Not a high-income country   No infants ≥34 week’s gestation  Control group not usual care   Other: ______________ |
| Notes: |

| **Study methods** | |
| --- | --- |
| Design |  Individually-randomized controlled trial   Cluster-randomized controlled trial |
| Unit of allocation |  Individual participant   Cluster/group - Specify:____________ |
| Number of centres |  Single centre study   Multicentre study  If multicentre, number of recruiting centres:­____________ |
| Country |  Australia  Canada  United States   Other:____________ |
| Notes: | |

| **Participant baseline characteristics**  If multiple treatment arms, only include the one most similar to eligibility criteria; multiple treatment groups to be pooled if similar. | | | |
| --- | --- | --- | --- |
| Characteristic | Control | Intervention | Total |
| Sample size (n) |  |  |  |
| Maternal (caregiver) age (years) – mean (SD) |  |  |  |
| Income (low-income if >75% of participants have low-income, as defined by authors) |  Low-income   Mixed income |  Low-income   Mixed income |  Low-income   Mixed income |
| Definition of low-income (include currency, if applicable): |  |  |  |
| Parity (primiparous if first child for 100% of participants) |  Primiparous   Primiparous and multiparous |  Primiparous   Primiparous and multiparous |  Primiparous   Primiparous and multiparous |
| Maternal Body Mass Index (overweight/obese if >75% of participants with BMI≥25.0 or as defined by study authors – use pre-pregnancy BMI, if available) |  Overweight/ obese   Other |  Overweight/ obese   Other |  Overweight/ obese   Other |
| Maternal overweight/obesity definition (e.g. pre-pregnancy BMI≥25.0): |  | | |
| Gestational age (term if 100% of participants born ≥37 weeks’ gestation – assumed term if no information) |  Term   Preterm and term |  Term   Preterm and term |  Term   Preterm and term |
| Race/Ethnicity – predominant race/ethnicity (% of participants who identify as such) |  |  |  |
| Notes: | | | |

| **Intervention** | |
| --- | --- |
| Brief description of intervention: (e.g. intervention provided to someone other than mother, components of intervention in addition to LC, asynchronous materials provided, curriculum, on-demand support, etc.): |  |
| Brief description of control/comparator: |  |
| Certification of LC (select all that apply) |  International Board Certified Lactation Consultant (IBCLC)   Certified Lactation Counselor (CLC)   Other:____________ |
| If provider not an IBCLC or CLC, list any training provided: |  |
| Timing |  Prenatal   Postnatal   Both |
| Provision |  In-person   Virtual   Both |
| Breastfeeding supplies (i.e. breast pump) |  Breast pump provided   Breast pump not-provided |
| Intensity – mean (SD) number of contacts by LC (i.e. text, call, visit, etc.) |  |
| Delivery |  Individual   Group   Both |
| Notes: | |

| **Outcomes** | | | | | | | | | | | | | | | |
| --- | --- | --- | --- | --- | --- | --- | --- | --- | --- | --- | --- | --- | --- | --- | --- |
| **Breastfeeding** | | | | | | | | | | | | | | | |
| Exclusive breastfeeding definition (if described): | |  | | | | | | | | | | | | | |
| Any breastfeeding definition (if described): | |  | | | | | | | | | | | | | |
| **1. Number of women who stop exclusive breastfeeding (EBF) before 6 months** | | | | | | | | | | | | | | | |
| Outcome measured |  Yes  No | | | | | | | | | | | | | | |
| Timepoint of measurement used (use measurement closest to 6mo but >6 weeks) |  | | | | | | | | | | | | | | |
| Results | Intervention | | | | | | | Control | | | | | | | |
|  | No. stopped EBF | | | No. still EBF | | Total | | No. stopped EBF | | | No. still EBF | | | Total | |
|  |  | | |  | |  | |  | | |  | | |  | |
| Notes (e.g. other results reported such as OR, RR, CI, p-value, etc.): | | | | | | | | | | | | | | | |
| **2. Number of women who stop any breastfeeding (BF) before 6 months** | | | | | | | | | | | | | | | |
| Outcome measured |  Yes  No | | | | | | | | | | | | | | |
| Timepoint of measurement used (use measurement closest to 6mo but >6 weeks) |  | | | | | | | | | | | | | | |
| Results | Intervention | | | | | | | Control | | | | | | | |
|  | No. stopped BF | | | No. still BF | | Total | | No. stopped BF | | | No. still BF | | | Total | |
|  |  | | |  | |  | |  | | |  | | |  | |
| Notes (e.g. other results reported such as OR, RR, CI, p-value, etc.): | | | | | | | | | | | | | | | |
| **3. Number of women who stop exclusive breastfeeding (EBF) before 4-6 weeks** | | | | | | | | | | | | | | | |
| Outcome measured |  Yes  No | | | | | | | | | | | | | | |
| Timepoint of measurement used (use measurement closest to ≤6weeks) |  | | | | | | | | | | | | | | |
| Results | Intervention | | | | | | | Control | | | | | | | |
|  | No. stopped EBF | | | No. still EBF | | Total | | No. stopped EBF | | | No. still EBF | | | Total | |
|  |  | | |  | |  | |  | | |  | | |  | |
| Notes (e.g. other results reported such as OR, RR, CI, p-value, etc.): | | | | | | | | | | | | | | | |
| **4. Number of women who stop any breastfeeding (BF) before 4-6 weeks** | | | | | | | | | | | | | | | |
| Outcome measured |  Yes  No | | | | | | | | | | | | | | |
| Timepoint of measurement used (use measurement closest to ≤6weeks) |  | | | | | | | | | | | | | | |
| Results | Intervention | | | | | | | Control | | | | | | | |
|  | No. stopped BF | | | No. still BF | | Total | | No. stopped BF | | | No. still BF | | | | Total |
|  |  | | |  | |  | |  | | |  | | | |  |
| Notes (e.g. other results reported such as OR, RR, CI, p-value, etc.): | | | | | | | | | | | | | | | |
| **5. Exclusive breastfeeding duration** | | | | | | | | | | | | | | | |
| Outcome measured |  Yes  No | | | | | | | | | | | | | | |
| Results | Intervention | | | | | | | Control | | | | | | | |
|  | n | | Mean | | SD | | | n | Mean | | | | SD | | |
|  |  | |  | |  | | |  |  | | | |  | | |
| Notes (e.g. other results reported such as mean difference (SD), n, median, IQR, range, CI, p-value, etc.): | | | | | | | | | | | | | | | |
| **6. Any breastfeeding duration** | | | | | | | | | | | | | | | |
| Outcome measured |  Yes  No | | | | | | | | | | | | | | |
| Results | Intervention | | | | | | | Control | | | | | | | |
|  | n | | Mean | | SD | | | n | Mean | | | | SD | | |
|  |  | |  | |  | | |  |  | | | |  | | |
| Notes (e.g. other results reported such as mean difference (SD), n, median, IQR, range, CI, p-value, etc.): | | | | | | | | | | | | | | | |
| **7. Maternal breastfeeding self-efficacy** | | | | | | | | | | | | | | | |
| Outcome measured |  Yes  No | | | | | | | | | | | | | | |
| Timepoint of measurement used **(**use time closest to 1 month postpartum but not before 2 weeks or after 6 weeks) |  | | | | | | | | | | | | | | |
| Tool used |  33 item Breastfeeding Self-efficacy Scale (BSES)   14 item BSES-Short Form (BSES-SF)   Other: ____________ | | | | | | | | | | | | | | |
| Results | Intervention | | | | | | | Control | | | | | | | |
|  | n | | Mean | | SD | | | n | Mean | | | | SD | | |
|  |  | |  | |  | | |  |  | | | |  | | |
| Notes (e.g. tool not validated; other results reported such as mean difference (SD), n, median, IQR, range, CI, p-value, etc.): | | | | | | | | | | | | | | | |
| **8. Infant overweight (OW)/obesity (OB)** | | | | | | | | | | | | | | | |
| Outcome measured |  Yes  No | | | | | | | | | | | | | | |
| How was OW/OB defined? (e.g. zBMI>2) |  | | | | | | | | | | | | | | |
| OW/OB reference population (if applicable – e.g. WHO, CDC, IOTF) |  | | | | | | | | | | | | | | |
| Timepoint of measurement used (use last available study measure) |  | | | | | | | | | | | | | | |
| Results | Intervention | | | | | | | Control | | | | | | | |
|  | No. OW/OB | | | No. not | | | Total | No. OW/OB | | | | No. not | | | Total |
|  |  | | |  | | |  |  | | | |  | | |  |
| Notes (e.g. other results reported such as OR, RR, CI, p-value, etc.): | | | | | | | | | | | | | | | |
| **9. Infant growth** | | | | | | | | | | | | | | | |
| Outcome measured |  Yes  No | | | | | | | | | | | | | | |
| Timepoint of measurement used **(**use last available study measure) |  | | | | | | | | | | | | | | |
| Growth measure (e.g. body mass index, weight for age, body fat mass, etc.): |  | | | | | | | | | | | | | | |
| Reference population (if applicable – e.g. WHO, CDC, IOTF) |  | | | | | | | | | | | | | | |
| Results | Intervention | | | | | | | Control | | | | | | | |
|  | n | | Mean | | SD | | | n | | Mean | | | SD | | |
|  |  | |  | |  | | |  | |  | | |  | | |
| Notes (e.g. other results reported such as mean difference (SD), n, median, IQR, range, CI, p-value, etc.): | | | | | | | | | | | | | | | |

*Add rows below for other infant growth measures

| **Miscellaneous** |
| --- |
| Notes: |
